# Glycolytic Reprogramming in Uterine Fibroids: Genetic, Transcriptomic, Proteomic, and Metabolomic Insights

**DOI:** 10.3390/genes16111268

**Published:** 2025-10-28

**Authors:** Samya El Sayed, Alvina Pan, Valentina Vanos, Rachel Michel, Mostafa Borahay

**Affiliations:** 1 Division of Reproductive Sciences and Women’s Health Research, Johns Hopkins School of Medicine, Baltimore, MD 21205, USA; 2 Department of Gynecology and Obstetrics, Johns Hopkins University School of Medicine, Baltimore, MD 21205, USA; 3 Geisel School of Medicine at Dartmouth, Hanover, NH 03755, USA

**Keywords:** uterine fibroids, metabolic reprogramming, glycolysis

## Abstract

Uterine leiomyomas or fibroids are a common but pernicious benign tumor impacting between 70–80% of women of reproductive age. Despite their high prevalence, the etiology of uterine fibroids is not fully understood. This review aims to highlight the distinct metabolic features that uterine fibroids adopt to meet biosynthetic demands, support proliferation, extracellular matrix production, survival, and fibrosis. Specifically, we posit the role of glycolytic reprogramming—an adaptation in fibrosis across organs (lung, kidney, heart, and liver) as a major contributor to uterine fibroid development. Previous genetic, transcriptomic, proteomic, and metabolic studies have drawn strong links between metabolism and uterine fibroid biology and identified genotype-specific metabolic alterations such as fumarate hydratase (FH) deficiency and mediator of RNA polymerase II transcription (MED12) gene mutations. Studies in non-uterine models have linked glycolysis to ECM production and fibrosis through activation of transforming growth factor-beta (TGF-β) and the canonical Wnt pathway (Wnt/β-catenin) signaling, supporting them as potential key pathways in uterine fibroid pathogenesis via glycolytic reprogramming. Other metabolic regulators, such as hypoxia-inducible factor 1-alpha (HIF-1α), mammalian target of rapamycin (mTOR), and phosphoinositide 3-kinase/protein kinase B (PI3K/Akt), may also sustain the fibrotic phenotype through coupling signaling that drives ECM production to metabolic programming. Overall, the proposed metabolic perspective of uterine fibroid pathogenesis invites further exploration of mechanistic investigation in uterine-specific models and therapeutic targeting through larger cohort studies.

## 1. Introduction

Uterine leiomyomas or fibroids are the most prevalent benign tumors of the uterus and arise in reproductive-aged women [1]. It is estimated that by age 50, over 70% of White women and over 80% of Black women will develop fibroids [2]. Black women are disproportionately affected, often experiencing an earlier onset of disease with larger and more numerous fibroids, contributing to a substantial symptomatic burden [3]. Common symptoms include prolonged, heavy, or irregular menstrual bleeding, pelvic pain or pressure, and abdominal fullness or bloating. Symptoms are clinically present in 25–50% of patients and vary based on fibroid size, number, and location [4]. Furthermore, fibroids constitute a significant psychosocial stressor and are linked to several patient-reported health disabilities, including poor quality of life [5]. Treatment options include pharmacologic therapy [6], radiologically guided interventions [7], and surgical options [8]. Notably, in many patients, medical management is inadequate, relegating patients to more invasive surgical solutions [9]. In fact, fibroids remain the leading indication for hysterectomy in the United States (US) and pose a significant burden to the national healthcare system, with combined direct and indirect costs estimated at 41.4 billion USD annually [10].

The development of uterine fibroids involves a combination of genetic, hormonal, and cellular factors. Genetic changes are thought to initiate tumor growth [11], while estrogen and progesterone drive continued enlargement during reproductive years [12]. Fibroids are also defined by excess extracellular matrix that increases both size and uterine stiffness [13]. More recently, stem cells in the myometrium have been implicated in sustaining fibroid growth [12]. Together, these processes underscore the complex biology and pathogenesis of fibroids and the continued difficulty of developing effective medical therapies.

Alongside these pathways, fibroids appear to adopt distinct metabolic features that help sustain their growth. One emerging concept is glycolytic reprogramming, a shift toward increased use of glycolysis for energy production when oxygen is available [14,15]. This adaptation supports both the energy demands and biosynthetic needs of rapidly growing tissue and may represent an underrecognized driver of fibroid biology.

## 2. Overview of Glycolytic Reprogramming

Metabolic reprogramming refers to a fundamental shift in cellular metabolism that enables cells to meet the increased bioenergetic and biosynthetic demands associated with rapid proliferation and survival under pathological conditions. Among these adaptations, glycolytic reprogramming, characterized by sustained reliance on glycolysis for energy production despite sufficient oxygen availability (the Warburg effect), has emerged as a hallmark of diverse disease states, including cancer and fibrotic disorders [14,15].

Under normal physiological conditions, glucose is efficiently metabolized through glycolysis, the tricarboxylic acid (TCA) cycle, and oxidative phosphorylation, yielding approximately 36–38 ATP molecules per glucose [14]. In contrast, cells undergoing glycolytic reprogramming preferentially convert glucose to lactate via aerobic glycolysis even under normoxic conditions, generating only ~2 ATP molecules per glucose [14]. Although energetically less efficient, this metabolic shift supports rapid ATP generation, supplies anabolic intermediates for macromolecular biosynthesis, and enhances cellular survival under stress.

**Figure 1:**
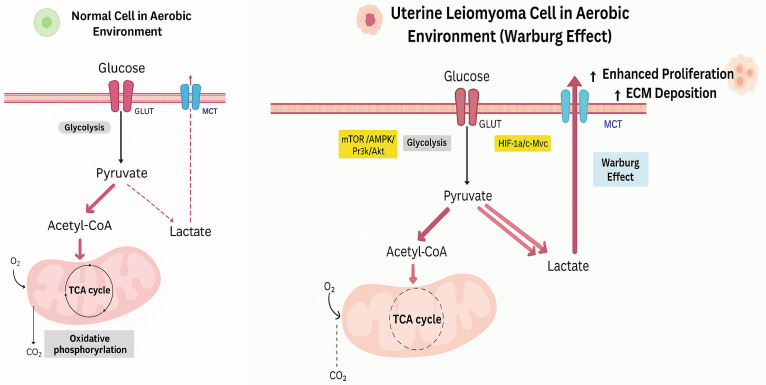
Schematic representation of the Warburg effect.

This reprogramming is governed by transcriptional and signaling networks. Key transcription factors include hypoxia-inducible factor-1α (HIF-1α) and c-Myc, which upregulate glucose transporters (e.g., GLUT1, GLUT4), and glycolytic enzymes such as hexokinase II and pyruvate kinase M2 (PKM2) [14,16,17]. Additional critical regulatory pathways, such as mTOR, AMP-activated protein kinase (AMPK), and PI3K/Akt signaling, modulate glycolytic flux in response to nutrient availability and cellular energy status [17,18] (Figure 1).

Beyond energy production, glycolytic reprogramming fuels critical biosynthetic and redox processes. Glycolytic intermediates are diverted into nucleotide, amino acid, and lipid synthesis pathways to support cell growth [15]. The pentose phosphate pathway generates NADPH for reductive biosynthesis and antioxidant defenses. Additionally, increased lactate production can acidify the extracellular environment, altering cell-cell signaling, immune cell infiltration, and matrix remodeling. These features collectively promote apoptosis resistance and stress adaptation.

Emerging evidence demonstrates that aerobic glycolysis is essential for establishing and maintaining the fibrotic phenotype across multiple organ systems. In pulmonary fibrosis, activated myofibroblasts exhibit enhanced glycolytic metabolism that supports collagen synthesis, proliferation, and apoptosis resistance [19]. Renal fibrosis is characterized by metabolic reprogramming in tubular epithelial cells and interstitial fibroblasts, with enhanced glycolytic flux driving tissue scarring and loss of kidney function [20]. Similarly, cardiac fibrosis following myocardial injury involves glycolytic activation in cardiac fibroblasts [21], while hepatic stellate cells in liver fibrosis shift towards glycolysis to sustain extracellular matrix production [22]. This consistent pattern across organ systems highlights glycolytic reprogramming as a conserved metabolic adaptation that enables cells to meet the substantial bioenergetic and biosynthetic demands of pathological matrix deposition and tissue remodeling.

Understanding this metabolic shift lays the foundation for exploring its role in uterine fibroid pathogenesis, where glycolytic reprogramming may contribute to the enhanced proliferation, extracellular matrix accumulation, and survival of fibroid cells, which are hallmarks of these clinically significant benign tumors.

## 3. Role of Glycolytic Reprogramming in Uterine Fibroids: What Is Currently Known?

### 3.1. Genetic Insights

Fumarate hydratase (FH) or fumarase is a key enzyme in the TCA cycle that catalyzes the hydration of fumarate to L-malate. The FH gene, located on chromosome 1q42.3–43, is a tumor suppressor composed of 10 exons spanning 22.15 kb of DNA. More than 130 pathogenic variants have been identified, most commonly missense mutations, followed by frameshift and nonsense variants [23]. These alterations can occur in both somatic and germline contexts and cause loss of function of the FH enzyme. FH mutations are present in approximately 0.4–1.6% [24,25,26] of all uterine fibroids, and the estimated carrier frequency of pathogenic FH variants in the general population is about 1 in 2563 individuals. Most FH-deficient uterine fibroids result from somatic mutations, while only 2.7–13.9% of affected women carry a germline variant [27]. Importantly, heterozygous germline variants in FH confer susceptibility to hereditary leiomyomatosis and renal cell cancer (HLRCC), a rare autosomal dominant condition characterized by early-onset cutaneous and uterine leiomyomas as well as renal cysts and tumors [28].

**Figure 2:**
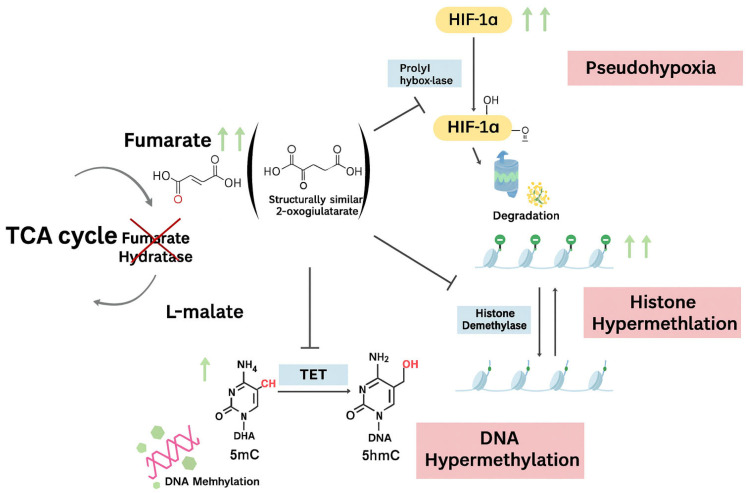
Schematic representation of fumarate hydratase’s role as an oncometabolite.

Downstream of FH loss, fumarate accumulates and functions as an oncometabolite with broad effects on cellular metabolism and gene regulation [29] (Figure 2). One of the most-characterized consequences is inhibition of prolyl hydroxylases, enzymes that normally hydroxylate HIF-1α and target it for degradation. When fumarate blocks this process, HIF-1α stabilizes and accumulates in normoxic conditions, creating a pseudohypoxic state [16,30]. Stabilized HIF-1α activates transcriptional programs that increase glucose uptake and glycolytic enzyme expression [16], which is a pattern consistent with aerobic glycolysis and reflected in the enrichment of glycolytic genes in FH-deficient fibroids [31,32]. HIF activity extends into adaptive responses such as angiogenesis and resistance to cell death, further promoting fibroid growth within the local environment [16]. Beyond this, fumarate can inhibit other α-ketoglutarate-dependent dioxygenases, including histone and DNA demethylases (e.g., TET DNA demethylases, JmjC-domain histone demethylases), adding an epigenetic factor to the subsequent pathway [29,33]. These downstream effects result in broader transcriptional and cellular changes distinguishing FH-deficient fibroids.

As follows, researchers have hypothesized that fibroids harboring an FH mutation would exhibit distinct gene expression profiles, specifically in genes related to energy homeostasis and glucose metabolism. Vanharanta et al. (2006) [31] (Table 1) tested this hypothesis by examining changes in global gene expression secondary to FH mutations. They performed transcriptome profiling using Affymetrix U133A oligonucleotide microarrays on FH-deficient fibroids (*n* = 7), FH-wild type fibroids (*n* = 15), and patient-matched myometrium samples (*n* = 11) from patients in a Finnish tertiary academic center to identify differentially expressed genes between FH-deficient and FH wild-type and myometrial samples. The FH-deficient fibroids included tissue from syndromic cases (truncating germline mutation [541delAG]; *n* = 5 from a patient with HLRCC) and non-syndromic cases (somatic missense mutation 586G > A [Ala196Thr], *n* = 1; somatic splice-site mutation IVS4 + 3A > G, *n* = 1). They found 297 differentially expressed genes between FH-deficient and FH-wild type fibroids. Of those, genes involved in hexose metabolism were the most significantly enriched in the FH-deficient fibroid group (*p* = 1.7 × 10^−9^), including all ten glycolytic enzymes (*p* = 1.1 × 10^−8^). Similarly, when comparing FH-mutant fibroids with normal myometrium, glycolytic enzymes were the most significantly upregulated in the fibroid group (*p* = 1.3 × 10^−7^) [31].

To further explore this, Catherino et al. (2007) [32] were interested in the molecular mechanisms maintaining energy homeostasis in FH-deficient uterine fibroids and investigated both glycolysis and Krebs cycle enzymes gene expression to identify potential compensatory effects. They measured differential gene expression using Affymetrix oligonucleotide microarrays (U133 chip), validated select targets with real-time reverse-transcription PCR on patient-matched fibroid and myometrial tissue samples from a patient with HLRCC (*n* = 3 specimens), and non-syndromic cases (*n* = 11). Specimens were obtained from individuals undergoing hysterectomy at a tertiary academic center in the United States. On microarray and cluster analysis of the tissues (using self-organizing map cluster analysis followed by dChip software analysis), the glycolysis cluster consistently showed altered expression. For example, fumarate hydratase was significantly downregulated in HLRCC fibroids compared to wild-type fibroids (measured as fold-change compared to matched-myometrium; *p* < 0.02), while several glycolysis enzymes were significantly upregulated, including hexokinase 1 (*p* < 0.01), phosphofructokinase (*p* < 0.01), aldolase (*p* < 0.01), triosephosphate isomerase (*p* < 0.01), phosphoglycerate kinase (*p* < 0.01), enolase, and pyruvate kinase (*p* < 0.01). Interestingly, however, there were no significant changes in the other Krebs cycle enzymes. Upon validation with real-time reverse-transcriptase PCR to quantitatively measure alterations in glycolysis enzymes and fumarate hydratase, results confirmed the microarray findings and showed that multiple glycolysis enzymes were overexpressed in HLRCC fibroids but not in nonsyndromic fibroids relative to patient-matched myometrium [32]. Notably, FH was underexpressed in both HLRCC fibroids and nonsyndromic fibroids, suggesting that FH downregulation may also occur in non-syndromic fibroids, warranting further investigation into metabolic alterations and adaptations of fibroids, even in the absence of FH mutations.

### 3.2. Transcriptomic Insights

Kwon et al. (2003) [34] conducted one of the earlier studies exploring differential gene expression in patient-matched fibroid-myometrium using cDNA analysis by DNA Chip. The researchers reported significantly greater expression of glycolysis enzymes, hexokinase 1 and hexokinase 2, in fibroid tissues related to myometrial tissues. Building on these initial findings, Alsamraae et al. (2025) [35] employed newer techniques such as whole-genome RNA sequencing (RNA-seq) to examine global gene expression and perform enriched pathway analyses of fibroid-myometrial tissue pairs among self-identified Black women (*n* = 6) in a United States tertiary academic center. The results supported the initial transcriptomic findings by demonstrating significant upregulation of the glycolysis pathway in fibroid tissue relative to myometrial tissue. Moreover, immunohistochemistry validation showed significantly greater monocarboxylate transporter 1 (MCT1) expression (a key transporter of lactate across cell membrane, facilitating lactate uptake and metabolism into cells under anaerobic conditions [39]; *p* < 0.05) coupled with significantly lower lactate dehydrogenase B (LDH-B) expression (an enzyme that catalyzes the reversible conversion of lactate to pyruvate with a greater affinity for lactate-to-pyruvate conversion under aerobic conditions [40]; *p* < 0.05) in fibroid tissue [35].

Taken together, these findings present preliminary evidence that glycolytic reprogramming plays a role in fibroid pathophysiology, even in the absence of FH mutation, to meet the metabolic and biosynthetic demands imposed by increased cellular proliferation and fibrosis.

### 3.3. Proteomic Insights

To further characterize molecular changes pertaining to glycolytic and other metabolic alterations in fibroids, Ura et al. (2016) [36] conducted a proteomic analysis to identify differentially expressed proteins in uterine fibroids compared to matched myometrial tissue. The researchers employed two-dimensional gel electrophoresis followed by mass spectrometry to analyze fibroid–myometrium tissue pairs from premenopausal women undergoing hysterectomy for symptomatic uterine fibroids in an Italian academic center (*n* = 8). Results demonstrated 24 protein spots that were significantly upregulated in fibroid tissue relative to myometrial tissue (defined as ≥1.5-fold change; *p* < 0.05). Of these, 12 proteins were linked to metabolic processes, including lipid metabolism, carbohydrate metabolism and oxidative phosphorylation, amino acid metabolism, nucleobase-containing compound metabolism, and protein metabolism. Specifically, in the context of glycolytic reprogramming, L-lactate dehydrogenase B chain (LDH-B) was upregulated in fibroid tissue (*p* = 0.014); this glycolytic enzyme catalyzes the reversible conversion of lactate to pyruvate and is involved in regenerating NAD^+^ from NADH. NAD+ is a cofactor for a number of glycolytic enzymes, and its regeneration is essential for sustaining glycolysis. Notably, these proteomic findings contrast with the previously mentioned transcriptomic and immunohistochemistry studies reporting lower LDH-B expression in fibroid tissue [36]. Such differences between RNA and protein abundance are not unexpected, as post-transcription and post-translational regulation can strongly influence enzyme stability and activity. This type of discrepancy has been observed in other disease contexts [41]. Rather than representing a true conflict, these findings suggest that LDH-B regulation in fibroids is context-dependent and multi-layered, with potential subtype-specific or microenvironmental influences.

Furthermore, the cytoplasmic isoform of malate dehydrogenase 1 (MDH1) was also significantly upregulated in fibroid tissue (*p* = 0.022) [36]. MDH1 catalyzes the conversion of oxaloacetate to malate while regenerating nicotinamide adenine dinucleotide (NAD+) from NADH in the process, effectively sustaining glycolysis. NAD+ plays a crucial role in glycolysis, in its capacity as an electron-carrier cofactor for glycolytic enzymes, including glyceraldehyde-3-phosphate dehydrogenase (GAPDH) and pyruvate kinase M2 (PKM2) [42]. Although LDH is classically recognized for regenerating NAD^+^ to allow glycolysis to continue, Hanse et al. (2017) [43] demonstrated that cytosolic MDH1 activity serves as a complementary mechanism for NAD^+^ regeneration, thereby promoting further glycolytic influx. Aspartate aminotransferase (GOT1) was also significantly increased (*p* = 0.018) [36]; this enzyme facilitates the reversible transamination between aspartate and α-ketoglutarate and contributes to the malate-aspartate shuttle, which further supports NAD+ regeneration.

### 3.4. Metabolomic Insights

To correlate molecular changes associated with glycolytic reprogramming to a metabolic phenotype, Duz et al. (2025) [37] employed a metabolomic approach to identify dysregulated metabolites and metabolic pathways in uterine fibroids. They collected fibroid and patient-matched myometrial tissue samples (designated as UF-MYM) from patients undergoing hysterectomy as definitive treatment for uterine fibroids at an academic center in Turkey (*n* = 15). Additionally, they collected control myometrial samples (designated as C-MYM) from patients undergoing hysterectomy for other benign gynecological conditions (*n* = 14). High-resolution magic angle spinning (HR-MAS) NMR spectroscopy was performed, followed by multivariate statistical analysis (principal component analysis (PCA) and partial least squares discriminant analysis (PLS-DA)) to assess global metabolic differences between cases and controls, and univariate analyses to identify individual metabolites contributing to the aggregate separation. The researchers reported a partial metabolic distinction between the fibroid samples and UF-MYM tissues, as evidenced by the PCA (cumulative R^2^ = 0.71) and PLS-DA (R^2^ = 0.172; Q^2^ = 0.05) score plots. Interestingly, there was a more pronounced separation among the UF-MYM compared to the control myometrium with C-MYM (cumulative R^2^ = 0.74) and PLS-DA (R^2^ = 0.169; Q^2^ = 0.127) score plots. On univariate analysis, several metabolites showed significant differences between tissue groups. For example, glutamine, glutamate, acetone, and lactate were present at significantly higher levels in fibroid samples relative to UF-MYM but at significantly lower levels in UF-MYM compared to C-MYM. Moreover, although glucose levels were not statistically different between the fibroid and UF-MYM group, the latter had significantly lower glucose levels compared to C-MYM. Collectively, these results are consistent with enhanced glycolytic activity in fibroids, coupled with metabolic adaptations that reduce reliance on glycolysis for TCA intermediates, instead upregulating alternative energy substrates, such as glutamine, to sustain TCA activity. While the functional role of glutamine metabolism in fibroids has not been well established, evidence from other proliferative and fibrotic contexts shows that collagen synthesis depends on glutamine utilization. Lung fibroblasts require glutamine for collagen protein production [44], and cancer-associated fibroblasts use glutamine-derived proline for collagen deposition in vivo [45]. These parallels suggest that glutamine utilization may support collagen biosynthesis and matrix accumulation in fibroids. Thus, glutamine metabolism may represent a shared metabolic dependency across fibroid subtypes.

Metabolomic evidence also points to metabolic heterogeneity in uterine fibroids, which preliminarily correlates with the presence of the major genetic drivers of fibroid pathogenesis: FH loss and MED12 mutation [46]. For instance, Heinonen et al. (2017) confirmed TCA cycle dysregulation in fibroids harboring FH mutations, alongside an increased flux in the pentose phosphate pathway. On the other hand, ascorbic acid metabolite levels were decreased in fibroids with MED12 mutations [38]. Notably, ascorbic acid is a cofactor for prolyl hydroxylase, an enzyme that synthesizes collagen for deposition into the extracellular matrix [47,48]. Moreover, ascorbic acid depletion may reflect its role in other cellular adaptations, such as epigenetic regulation through its role as a cofactor for DNA and histone demethylases [47]. In contrast, other metabolic aberrations were consistent across all fibroid subtypes, such as altered heme metabolism [38]. Heme is an essential cofactor for cellular respiration and ATP production [49]. Importantly, increased influx through the heme metabolism pathway promotes the removal of accumulated TCA metabolites, a process particularly relevant in FH-deficient fibroids [50]. However, its role in other fibroid subtypes is less clear. Nevertheless, altered heme metabolism warrants further investigation and may represent a potential therapeutic target across leiomyoma subtypes.

These genetic, transcriptomic, proteomic, and metabolic data suggest that glycolytic reprogramming is not limited to the rare FH-deficient subtype but extends to more common fibroid variants, including those harboring MED12 mutations and HMGA2 overexpression. Heinonen et al. demonstrated that altered metabolite profiles and disrupted heme and ascorbate metabolism were shared across MED12 and wild-type fibroids, indicating a broader metabolic phenotype [38]. These converging multi-omics findings indicate that glycolytic reprogramming likely represents a generalizable driver of fibroid pathophysiology across genetic backgrounds, supporting proliferation, extracellular matrix accumulation, and survival.

## 4. Mechanistic Links Between Glycolytic Reprogramming and Fibroid Pathogenesis

### 4.1. Transforming Growth Factor—Beta (TGFβ) Activation

Transforming growth factor-beta (TGFβ) is a family of ubiquitously expressed multifunctional cytokines involved in regulating several cellular processes, including proliferation, differentiation, fibrosis [51], senescence, and apoptosis [52] through complex autocrine and paracrine signaling [53]. It is a dimeric polypeptide with three distinct isoforms (TGF-β1, TGF-β2, and TGF-β3) [54] that resides in its latent form in the extracellular matrix (ECM) [55] and may be activated by interactions with cell surface receptors (such as integrin) [56], enzymes (such as matrix metalloproteinases (MMP)) [57] or physical and chemical changes in the cellular microenvironment (such as increases in shear stress or in pH) [58]. Once activated, it subsequently binds to corresponding cell surface receptors (mainly TGF-βR-I and TGF-βR-II), inducing several downstream signaling cascades that regulate the transcriptional activity of many genes involved in tissue homeostasis and development [59]. These pathways are broadly categorized into a canonical Smad pathway and other Smad-independent pathways, including PI3K/Akt/mTOR, Ras/Raf/MEK/ERK, and FAK [58], and have been implicated in the establishment and maintenance of the fibrotic phenotype through the promotion of myofibroblast activation and ECM production [60]. In fact, TGF-β has been recognized as the primary driver of fibrosis in a number of fibrotic diseases of the cardiac [61], renal [62], and hepatic systems [63].

Accumulating evidence over the past decades has highlighted the role of TGF-β, specifically the TGF-β3 isoform, as an important player in fibroid pathogenesis. For instance, Lee and Nowak (2001) [64] reported higher levels of TGF-β3 mRNA and protein expression in leiomyoma cells as compared to autologous myometrial cells. Interestingly, the researchers also reported that leiomyoma cells exhibit a refractory response to the pro-apoptotic, anti-proliferative effect of TGF-β. Similarly, Norian et al. (2009) [65] demonstrated that TGF-β mRNA transcripts were increased in leiomyoma cells as compared to myometrial cells and that TGF-β signaling promotes the production of versican, a critical ECM protein, specifically increasing the production of glycosaminoglycan (GAG) rich-versican variants and contributing to the excessive, disordered ECM characteristic of uterine fibroids. Furthermore, Jospeh et al. (2010) found that TGF-β3 stimulation induces a fibroid-like molecular phenotype in myometrial cells by upregulating expression of ECM-deposition related genes (such as collagen and fibronectin) and decreasing expression of ECM-degradation related genes (such as MMP 2 and MMP 11) [66]. Clinically, elevated serum concentrations of TGF-β3 were associated with higher odds of developing uterine fibroids [67].

Beyond its established role in promoting fibrosis and ECM accumulation, TGF-β signaling is increasingly recognized as a regulator of cellular metabolism, particularly glycolytic reprogramming. The first evidence linking TGF-β to glucose metabolism emerged from studies in Swiss mouse 3T3 fibroblasts, where TGF-β treatment increased GLUT1 mRNA levels and stimulated glucose uptake [68]. This relationship is bidirectional: high glucose promotes TGF-β ligand production, increases TβRI/TβRII availability, and facilitates latent TGF-β activation via matrix metalloproteinases, amplifying downstream Akt–mTOR signaling and promoting fibroblast hypertrophy [69].

TGF-β enhances glycolysis through multiple mechanisms. In cancer and fibrotic models, TGF-β1 induces GLUT1 overexpression, which correlates with epithelial–mesenchymal transition (EMT) markers and increased glucose uptake [70]. It also upregulates glycolytic enzymes such as HK2 and PFKFB3, driving glycolytic flux, lactate production, and invasive potential [71]. Constitutive activation of TβRI in fibroblasts is sufficient to induce the Warburg effect, enabling metabolic support of neighboring cells [72]. To date, there is no direct fibroid-specific evidence demonstrating that glycolytic enzymes such as HK2 or PFKFB3 mechanistically regulate TGF-β signaling or ECM deposition. However, multiple transcriptomic datasets confirm upregulation of HFK2 and PFKFB3 in fibroid tissue compared to myometrium [34,35], and TGF-β3 expression is consistently elevated in leiomyoma cells. These parallels suggest that similar mechanisms are likely operative in fibroids, though fibroid-specific functional studies remain a critical gap.

**Figure 3:**
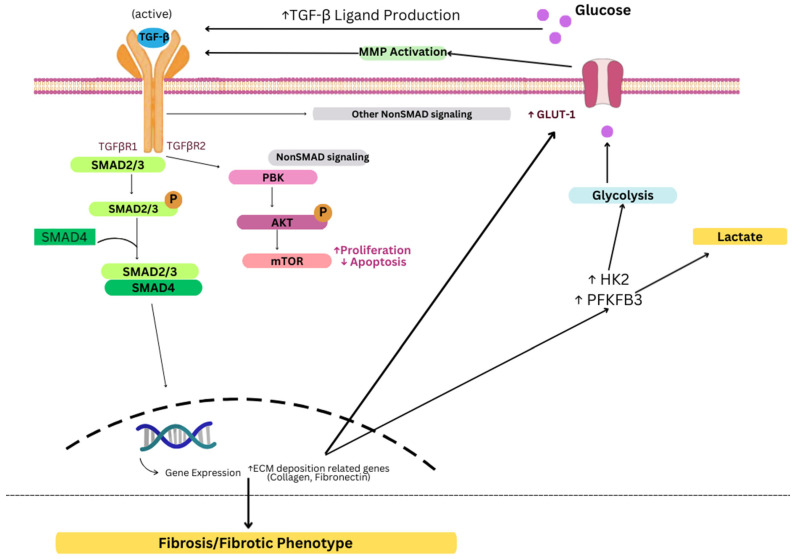
Schematic representation of the TGF-β–Warburg effect–lactate–TGF-β loop.

Furthermore, lactate generated through glycolysis reinforces TGF-β signaling by acidifying the extracellular space, activating latent TGF-β, and promoting EMT [73]. This establishes a “TGF-β–Warburg effect–lactate–TGF-β” loop that sustains both metabolic and fibrotic activity (Figure 3).

Although most evidence comes from cancer and other fibrotic models, parallels with fibroids are compelling. Fibroid cells exhibit elevated TGF-β3 expression [64,65] and increased glycolytic enzyme expression [34,35]. Together, these findings suggest that TGF-β-driven glycolytic reprogramming in fibroids may simultaneously support proliferation and excessive ECM deposition, reinforcing their pathophysiologic persistence.

### 4.2. Wnt/β Catenin Signaling

The Wingless-type MMTV integration site family (Wnt) signaling pathway is an evolutionarily preserved signal pathway that encodes several biological functions, including cellular proliferation, developmental migration, stem cell maintenance, and tissue homeostasis [74]. Importantly, TGF-β activation can act upstream by stimulating the secretion of Wnt proteins into the ECM [75,76]. Once secreted, they can bind transmembrane cell surface receptors such as Frizzled 1–10 (FZD 1–10) and LPR5/6 to initiate a cascade of intracytoplasmic and intranuclear signaling pathways, broadly grouped into canonical (β-catenin-dependent) [77] and non-canonical (β-catenin independent) pathways [78]. In the canonical pathway, β-catenin translocates to the nucleus to act as a transcriptional co-activator, facilitating expression of target genes involved in cell survival, differentiation, and migration [74]. As such, aberrant Wnt/β-catenin signaling has been established as a mechanism instrumental to tumorigenesis [79] and has more recently garnered momentum as a process central to fibrosis and is implicated in several fibrotic diseases of the cardiac [80], pulmonary [81,82], and renal [83] systems.

A growing body of evidence has evaluated the role of Wnt/β-catenin in fibroid pathophysiology. For instance, Mangioni et al. (2005) reported higher rates of Wnt ligands (such as Wnt5a) in primary human leiomyoma cells relative to their myometrial counterparts [84]. Moreover, Ono et al. (2013) [85] uncovered an underlying mechanism of the Wnt/β-catenin signaling in leiomyogenesis through crosstalk with estrogen/progesterone-dependent pathways of leiomyoma stem/progenitor cells (LMSP). Estrogen/progesterone treatment of myometrial cells increased production of Wnt ligands (Wnt11 and Wnt16) and subsequently sent paracrine mitogenic signals to neighboring LMSP, which in turn, induced β-catenin translocation and transcriptional regulation, resulting in LMSP proliferation and leiomyoma cell propagation. Interestingly, El Sabeh et al. (2021) [86] presented evidence for mechanistic links between MED12 mutations and canonical Wnt/β-catenin signaling, whereby absent or deficient MED12 promotes β-catenin-dependent signaling and growth in uterine fibroids.

Evidence also increasingly links Wnt/β-catenin activation to glycolytic reprogramming. In colon cancer models, Wnt signaling directly induces pyruvate dehydrogenase kinase 1 (PDK1) transcription via β-catenin/TCF binding to its promoter [87]. PDK1 phosphorylates and inactivates the pyruvate dehydrogenase (PDH) complex, blocking pyruvate entry into the tricarboxylic acid (TCA) cycle and shunting it toward lactate production, a hallmark of the Warburg effect. Wnt also upregulates lactate dehydrogenase A (LDH-A) and monocarboxylate transporter-1 (MCT-1), facilitating lactate production and export [87]. These effects collectively enhance aerobic glycolysis while suppressing oxidative phosphorylation.

**Figure 4:**
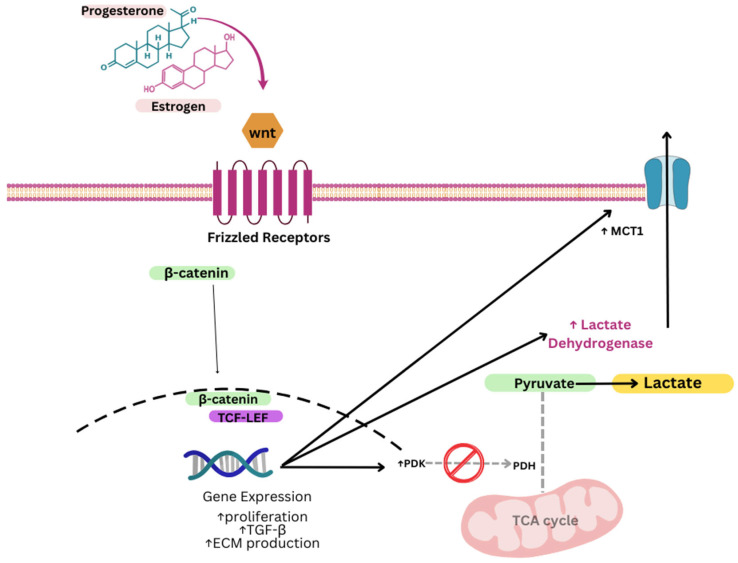
Schematic representation of downstream Wnt signaling leading to lactate accumulation.

Metabolic reprogramming downstream of Wnt signaling involves both short-term and long-term regulatory modes. Short-term effects include mTORC2-Akt-mediated increases in glycolytic enzyme stability within minutes of Wnt stimulation, independent of β-catenin transcriptional activity [88]. Long-term effects rely on β-catenin-dependent transcription of metabolic enzymes and transporters, establishing a sustained glycolytic phenotype [87]. This phenotype is reinforced by Wnt-induced HIF-1α stabilization, which further promotes glycolytic gene expression and lactate accumulation [89]. Lactate, in turn, can modulate the tumor or fibrotic microenvironment, supporting angiogenesis and further sustaining Wnt signaling (Figure 4).

Although direct studies in uterine fibroids are lacking, Wnt/β-catenin signaling is upregulated in fibroid tissue and is involved in fibroid stem cell proliferation and ECM production [84,85]. Given its established ability to drive glycolysis in other proliferative contexts, Wnt/β-catenin signaling may represent a mechanistic link between glycolytic reprogramming and fibroid pathogenesis—promoting both the high proliferative capacity of fibroid cells and the biosynthetic demands of excessive ECM deposition.

### 4.3. Extracellular Matrix Stiffness and Mechanotransduction

Uterine fibroids are characterized not only by smooth muscle proliferation but also by extensive extracellular matrix (ECM) accumulation, contributing significantly to their size, stiffness, and clinical symptoms [90,91]. Compared to adjacent myometrium, fibroids exhibit markedly increased levels of collagen types I and III, fibronectin, and proteoglycans, resulting in a disorganized, fibrotic ECM that imparts significantly higher tissue stiffness [92,93]. This altered ECM architecture is not merely a byproduct of fibroid growth; it is a dynamic regulator of cell behavior, activating a variety of signaling cascades through mechanotransduction [93,94].

Mechanotransduction refers to the process by which cells sense and respond to mechanical cues such as matrix stiffness, fluid shear stress, or tension. Transmembrane receptors such as integrins and CD44 serve as mechanosensors, relaying information about the ECM to intracellular networks including focal adhesion kinase (FAK), Rho/ROCK, PI2K, Akt, and Hippo pathway effectors like Yes-associated protein (YAP) and transcriptional coactivator with PDZ-binding motif (TAZ) [92,95]. These mechanosensitive pathways are increasingly recognized not only for their roles in regulating fibrotic gene expression and survival, but also for their capacity to reprogram cellular metabolism.

In particular, YAP/TAZ activation in response to ECM stiffness has been shown to upregulate glycolytic gene expression across a variety of proliferative systems. Nuclear translocation of YAP/TAZ induces transcription of key metabolic regulators, including GLUT1 (glucose transporter), HK2 (hexokinase 2), and LDHA (lactate dehydrogenase A), driving enhanced glycolytic flux and favoring the Warburg phenotype [95,96,97]. This metabolic rewiring is further supported by stiffness-induced stabilization of HIF-1α even under normoxic conditions, reinforcing glycolytic enzyme expression and suppressing mitochondrial oxidative phosphorylation [94]. Additional studies have shown that mitochondrial fragmentation and shifts in redox state are downstream of ECM stiffness, further pushing cells toward aerobic glycolysis.

Although direct studies in uterine fibroids examining this pathway are limited, these findings collectively suggest that mechanotransduction—especially via YAP/TAZ—may serve as a key link between ECM stiffness and glycolytic reprogramming. Given the established upregulation of YAP/TAZ signaling in fibroid tissue along with their dense and highly crosslinked ECM, we propose that stiff fibroid matrices contribute to metabolic adaptation via this pathway [98]. This mechanochemical reprogramming would enable fibroid cells to meet the increased bioenergetic and biosynthetic demands of sustained proliferation and ECM production, parallel to other fibrotic tumor models. The fibrotic ECM in fibroids thus serves as both a structural and signaling platform, influencing cell survival, proliferation, and metabolic phenotype.

### 4.4. Pathway Crosstalk and Synergistic Effects

While TGF-β, Wnt/β-catenin, and mechanotransduction pathways each likely contribute to glycolytic reprogramming in fibroids, their interactions and synergistic effects likely amplify metabolic rewiring beyond what individual pathway activation could achieve. These pathways form an interconnected network where activation of one pathway can enhance and sustain the others, creating feed-forward loops that perpetuate both fibrotic and metabolic dysfunction in fibroids.

All three pathways converge on key nodes of glycolytic regulation, allowing for synergistic metabolic reprogramming. HIF-1*α* represents a critical convergence point, stabilized by TGF-β signaling, Wnt activation, and ECM stiffness [73,89,94]. This triple regulation may result in robust glycolytic gene expression exceeding what any single pathway could achieve. Similarly, these pathways collectively target multiple glycolytic steps: TGF-β upregulates HK2 and PFKFB3; YAP/TAZ promotes GLUT1, HK2, and LDHA; and Wnt enhances PDK1 and lactate production. Importantly, the ECM serves as both a product and regulator of these pathways, functioning as a hub for central integration. ECM proteins create the mechanical environment activating mechanotransduction, while lactate from glycolytic reprogramming acidifies the ECM, promoting further TGF-β activation in a self-reinforcing cycle. In this sense, fibroids represent a pathological state where mechanical, biochemical, and metabolic signals are locked in mutually reinforcing loops.

## 5. Clinical and Therapeutic Implications

Glycolytic reprogramming represents a potential contributor to the clinical manifestations of fibroids. As previously described, glycolytic reprogramming can fuel extracellular matrix production. Fibroids contain approximately 50% more ECM than adjacent myometrium, and the resulting stiffness contributes to tumor growth and progression [99]. These structural changes contribute to heavy menstrual bleeding, pelvic pain, and uterine enlargement [100,101]. Submucous fibroids can additionally impair fertility through distortion of the uterine cavity [8]. By sustaining extracellular matrix production and stiffening, glycolytic reprogramming may directly exacerbate clinical symptoms such as heavy bleeding, pelvic pain, and infertility. Moreover, by maintaining proliferative and fibrotic signaling independently of estrogen and progesterone, glycolysis may contribute to treatment resistance, echoing mechanisms of hormone therapy escape described in other hormone-dependent tumors [102,103].

Given these clinical consequences, pathways that sustain glycolytic reprogramming may also represent promising therapeutic targets. Glycolytic reprogramming in fibroids is closely tied to hypoxia-responsive pathways, where transcriptional regulation is largely mediated by HIF-1α [104]. HIF-1α itself is a strong regulator of glycolysis, and angiogenesis has also been implicated in ECM production in fibrotic disease contexts [16,105]. Direct pharmacologic inhibition of HIF-1α has been studied in solid tumors such as pancreatic and prostate cancer. The HIF-1α inhibitor PX-478 suppresses glycolysis and angiogenesis in preclinical models and has started early-phase clinical evaluation in advanced malignancies [106,107]. These findings provide a precedent for suggesting that targeted HIF-1 α could represent a strategy to disrupt glycolytic reprogramming in fibroids, though this context has not been investigated.

In addition to broadly relevant regulators like HIF-1α, genotype-specific metabolic alterations may also provide therapeutic opportunities. For example, FH-deficient fibroids show heightened activation of the pentose phosphate pathway (PPP) and alterations offer therapeutic opportunities [38]. Glucose-6-phosphate dehydrogenase (G6PD) functions as one of the key rate-limiting enzymes in the PPP and has been studied as a therapeutic drug target. Pre-clinical oncology models have shown that pharmacological and genetic inhibition of G6PD reduces proliferation and disrupts NADPH-dependent redox metabolism [108,109]. This strategy has not been investigated in the fibroid context, but the heightened PPP activation suggests that targeting G6PD could represent another method of disrupting glycolytic reprogramming that contributes to fibrosis.

## 6. Conclusions

Evidence across genetic and molecular studies shows that uterine fibroids exhibit glycolytic reprogramming, a consistent feature that supports both cell growth and the buildup of extracellular matrix (ECM). This metabolic shift connects directly to well-known drivers of fibrosis, including TGF-β, Wnt/β-catenin, and YAP/TAZ signaling. Together, these interactions create a self-reinforcing cycle in which altered metabolism and fibrosis continually strengthen one another. This perspective indicates that metabolism is not merely a secondary effect but is directly involved in the processes that sustain fibroid growth. In practice, this highlights the potential for treatments that target glycolytic mechanisms, for example, by inhibiting lactate export, reducing pentose phosphate pathway activity in FH-deficient tumors, or combining metabolic and hormonal therapies. By framing glycolytic reprogramming as a core part of fibroid biology, future studies can move beyond hormone-centered models to include metabolic and other non-hormonal drivers. This approach opens the possibility of therapies that interrupt the metabolic–fibrotic loop, with the potential to reduce tumor growth and associated symptoms.

## Figures and Tables

**Table 1:** Overview of Multi-Omics Approaches to Glycolytic Reprogramming in Uterine Fibroids.

Approach	Study (Year)	Objective	Biological Model	Analytic Methods	Results
Genetic	Vanharanta et al. (2006) [31]	To determine the differences in global gene expression in FH mutant fibroids vs. wild-type fibroids	Fresh-frozen myometrium (*n* = 11) Fresh-frozen fibroids (*n* = 22): - Sporadic FH mutations (*n* = 2) - Germline FH mutations (*n* = 5) - Wild-type FH (*n* = 15)	Microarray (GeneChip^®^ HG-U133A, TIGR MeV); validation with qRT-PCR	↑ Expression of all ten differentially expressed genes involved in glycolysis in FH mutants compared FH wild-type fibroids and myometrial tissue
	Catherino et al. (2007) [32]	To determine gene expression of glycolysis and TCA cycle enzymes in HLRCC fibroids vs. non-syndromic fibroids	Matched human fibroid- myometrium pairs from patients with HLRCC (*n* = 1; 3 samples) and without HLRCC (*n* = 11)	Microarray (Affymetrix U-133 chip)	Non-syndromic fibroids No differential expression in glycolysis or TCA cycle enzymes compared to matched myometrium HLRCC fibroids ↓ Fumarate hydratase ↑ Glycolysis enzymes compared to matched myometrium
Transcriptomic	Kwon et al. (2003) [34]	To investigate differential gene expression between fibroid and myometrial tissue	Matched fibroid-myometrium pairs (*n* = 5)	DNA microarray/chip; validation with RT-PCR	↑ Hexokinase 1 and ↑ Hexokinase 2 expression (>3 fold) in fibroid tissue vs. myometrial tissue
	Alsamraae et al. (2025) [35]	To investigate differential global genomic expression profiles gene expression between fibroid and myometrial tissue	Matched fibroid-myometrium pairs (*n* = 5)	Whole-genome RNA sequencing (RNA-seq), pathway enrichment; validation with IHC	↑ glycolysis pathway activity (>50-fold enrichment) IHC ↑ MCT1 expression ↓ LDHB expression in fibroid tissue compared to myometrium
Proteomic	Ura et al. (2016) [36]	To	Matched fibroid-myometrium pairs (*n* = 5)	2-DE + mass spectrometry (MS); validation with Western blotting	Upregulation of LDH-B, MDH-1, and GOT-1
Metabolomic	Duz et al. (2025) [37]	To determine dysregulated metabolites in fibroid vs. myometrial tissues	Matched fibroid-myometrium pairs (*n* = 5) Control myometrium samples (*n* = 14)	HR-MAS NMR; multivariate analysis (PCA, PLS-DA); validation with univariate analysis	Fibroids vs. adjacent myometrium ↑ Lactate, alanine, glutamate, glutamine, methionine, isocitrate, choline, GPC, PC, o-phosphoethanolamine, taurine, myo-inositol, phenylacetate, ascorbate, glucose, methylhistidine Fibroid-adjacent myometrium vs. control myometrium ↓ Valine, leucine, isoleucine, ethanol, arginine, N-acetyl tyrosine, acetone, p-methylhistidine, glucose, phenylacetate, myo-inositol, α-glucose
	Heinonen et al. (2017) [38]	To determine dysregulated metabolites in fibroid subtypes (MED12, HMGA2, FH)	Fibroid samples (*n* = 25; from 17 patients) FH-deficient (*n* = 7) MED12 (*n* = 7) HMGA2 overexpression (*n* = 2) Triple wild-type fibroids (*n* = 9) Myometrium samples (*n* = 17) Matched controls	LC-MS/MS; pathway enrichment	Shared across subtypes ↓ heme and homocarnosine FH-deficient subtype ↑ TCA intermediates (fumarate, malate, succinate, α-KG), PPP activation (↑ G6PD/PGD/TKT), unique ↑ N6-succinyladenosine and argininosuccinate. MED12 subtype ↓ vitamin A, ↓ vitamin C metabolites, ↓ multiple amino acids and sphingolipids; altered methionine/cysteine/SAM/taurine metabolism. HMGA2 overexpression subtype Distinct but less pronounced lipid/amino acid changes; separate clustering from FH/MED12

**Abbreviations: FH**, fumarate hydratase; **HLRCC**, hereditary leiomyomatosis and renal cell carcinoma; **qRT-PCR**, quantitative real-time polymerase chain reaction; **RT-PCR**, reverse transcription polymerase chain reaction; **RNA-seq**, RNA sequencing; **IHC**, immunohistochemistry; **2-DE**, two-dimensional gel electrophoresis; **MS**, mass spectrometry; **HR-MAS NMR**, high-resolution magic angle spinning nuclear magnetic resonance; **PCA**, principal component analysis; **PLS-DA**, partial least squares discriminant analysis; **LC-MS/MS**, liquid chromatography–tandem mass spectrometry; **PPP**, pentose phosphate pathway; **GPC**, glycerophosphocholine; **PC**, phosphocholine; **TKT**, transketolase; **SAM**, S-adenosylmethionine.

## Data Availability

No new data were created or analyzed in this study.
